# The In Vitro Non-Tetramerizing ZapA^I83E^ Mutant Is Unable to Recruit ZapB to the Division Plane In Vivo in *Escherichia coli*

**DOI:** 10.3390/ijms21093130

**Published:** 2020-04-29

**Authors:** Nils Y. Meiresonne, Tanneke den Blaauwen

**Affiliations:** Bacterial Cell Biology and Physiology, Swammerdam Institute for Life Sciences, Faculty of Science, University of Amsterdam, Science Park 904, 1098 XH Amsterdam, The Netherlands; n.y.meiresonne@uva.nl

**Keywords:** Cell division, *ter* linkage, MinC, Z-associated protein A (ZapA), Z-associated protein B (ZapB), filamenting temperature-sensitive Z (FtsZ)

## Abstract

Bacterial cell division is guided by filamenting temperature-sensitive Z (FtsZ) treadmilling at midcell. FtsZ itself is regulated by FtsZ-associated proteins (Zaps) that couple it to different cellular processes. Z-associated protein A (ZapA) is known to enhance FtsZ bundling but also forms a synchronizing link with chromosome segregation through Z-associated protein B (ZapB) and *matS-*bound MatP. ZapA likely exists as dimers and tetramers in the cell. Using a ZapA mutant that is only able to form dimers in vitro (ZapA^I83E^), this paper investigates the effects of ZapA multimerization state on its interaction partners and cell division. By employing fluorescence microscopy and Förster resonance energy transfer in vivo it was shown that ZapA^I83E^ is unable to complement a *zapA* deletion strain and localizes diffusely through the cell but still interacts with FtsZ that is not part of the cell division machinery. The diffusely-localized ZapA^I83E^ is unable to recruit ZapB, which in its presence localizes unipolarly. Interestingly, the localization profiles of the chromosome and unipolar ZapB anticorrelate. The work presented here confirms previously reported in vitro effects of ZapA multimerization in vivo and places it in a broader context by revealing the strong implications for ZapB and chromosome localization and *ter* linkage.

## 1. Introduction

The cell division protein Z-associated protein A (ZapA) is broadly conserved among Gram-negative and -positive bacteria [1,2]. In *Escherichia coli* ZapA promotes filamenting temperature-sensitive Z (FtsZ) polymerization through enhancing cooperativity of FtsZ polymer association [3]. However, the turnover of FtsZ is highly dynamic and its filaments would thus not benefit from being rigid when constriction occurs [4]. Many of the ZapA-enhanced FtsZ polymerization studies were performed in vitro at non-physiological conditions that themselves promote FtsZ filamentation and bundling [2]. Experiments performed under physiological conditions revealed a more dynamic stabilizing effect of ZapA on FtsZ bundle formation [3]. 

An outstanding question is whether the proposed stabilizing effects of ZapA influence FtsZ treadmilling dynamics. Very recently, in vitro work showed that transient interactions of ZapA with FtsZ increase the spatial order and stabilize the architecture of the FtsZ filament network without affecting its treadmilling velocity [5]. These effects were only observed for ZapA that was able to interact with FtsZ [5]. ZapA can exist as a mixture of dimers and tetramers in vitro but in vivo it is likely to be mostly tetrameric due to molecular crowding conditions [3,6,7]. In fact, available ZapA structures and in vitro cross-linking suggest a tetrameric structure, which is thought to be required for FtsZ bundling [6,8,9]. These in vitro results prompt an in vivo explanation of ZapA multimerization functionality. ZapA interacts with FtsZ as a component of the chromosome replication terminus (*ter*) linkage that synchronizes cell division with chromosome segregation [10]. In *ter* linkage, ZapA interacts with Z-associated protein B (ZapB), which interacts with MatP that binds 23 *matS* sequences distributed on the *ter* domain and condenses this region of the chromosome [11,12,13]. Therefore, ZapA multimerization dynamics should be investigated in the context of both cell division and chromosome segregation. 

Here we describe the molecular behavior of ZapA and its interacting proteins in vivo. The in vitro non-tetramerizing mutant ZapA^I83E^ was unable to complement the *zapA* deletion phenotype. Although it did not specifically localize to the division site, it was able to interact with FtsZ elsewhere in the cell, titrating some of it away from midcell. ZapB midcell localization was changed in cells that express ZapA^I83E^ but did not show the same diffuse localization pattern. Instead, it resided predominantly at one cell pole confirming that ZapA midcell localization is important for ZapB interaction. Furthermore, we present that chromosomal localization patterns anticorrelate with polar ZapB in cells without ZapA or with the in vitro non-tetramerizing mutant ZapA^I83E^, resulting in asymmetric cell division.

## 2. Results

### 2.1. ZapA^I83E^ Does Not Complement the ∆zapA Phenotype

**Figure 1:**
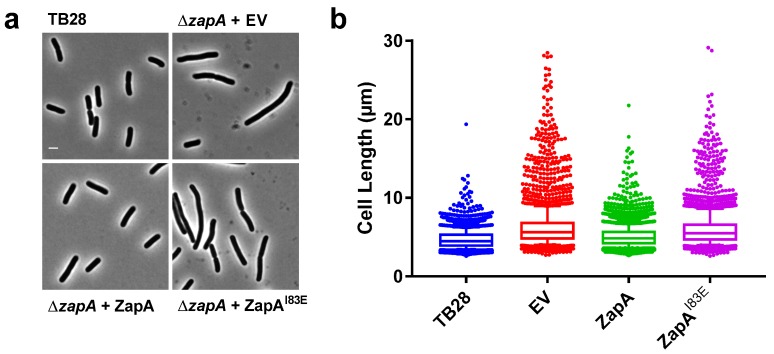
ZapA^I83E^ does not complement the elongated ∆*zapA* phenotype. (**a**) Phenotypes of the cells. The scale bar represents 2 µm. (**b**) Cell length distributions of each group. The number of cells and their average length measured were for the wild-type parental strains TB28; 3670 and 4.5 μm, EV; 2686 and 6.4 μm, from plasmid-expressed ZapA^WT^; 4476 and 5.1 μm and ZapA^I83E^; 2491 and 6.1 μm, respectively. The whiskers represent the 10th and 90th percentile.

The work of Pacheco-Gómez et al. [6] shows that ZapA tetramerization is required for in vitro FtsZ bundling using ZapA mutant I83E that only forms dimers. This mutant fully folds and forms ZapA dimers that were shown to still bind FtsZ by co-sedimentation [6]. To assess whether ZapA^I83E^ would complement the elongated ∆*zapA* phenotype and restore wild-type morphology in vivo, a complementation experiment was performed. Expression of wild-type ZapA, ZapA^I83E^ or a negative empty vector (EV) control was induced from plasmid with 50 µM isopropyl β-D-1-thiogalactopyranoside (IPTG) in TB28 ∆*zapA* cells growing in rich medium for ~8 mass doubling as described before [7]. The cells were then fixed, imaged, and average cell lengths were analyzed. This showed that ZapA^I83E^ was unable to complement ∆*zapA* with 6% of the cells being longer than 10 µm compared to wild-type ZapA with 1% of cells longer than 10 µm. ZapA^I83E^ results resembled more the EV control, which had 8% of the cells longer than 10 µm and ZapA results resembled more the TB28 parental strain with 0.5% of cells longer than 10 µm (Figure 1). 

### 2.2. ZapA^I83E^ Localizes Diffusely Throughout the Cell

**Figure 2:**
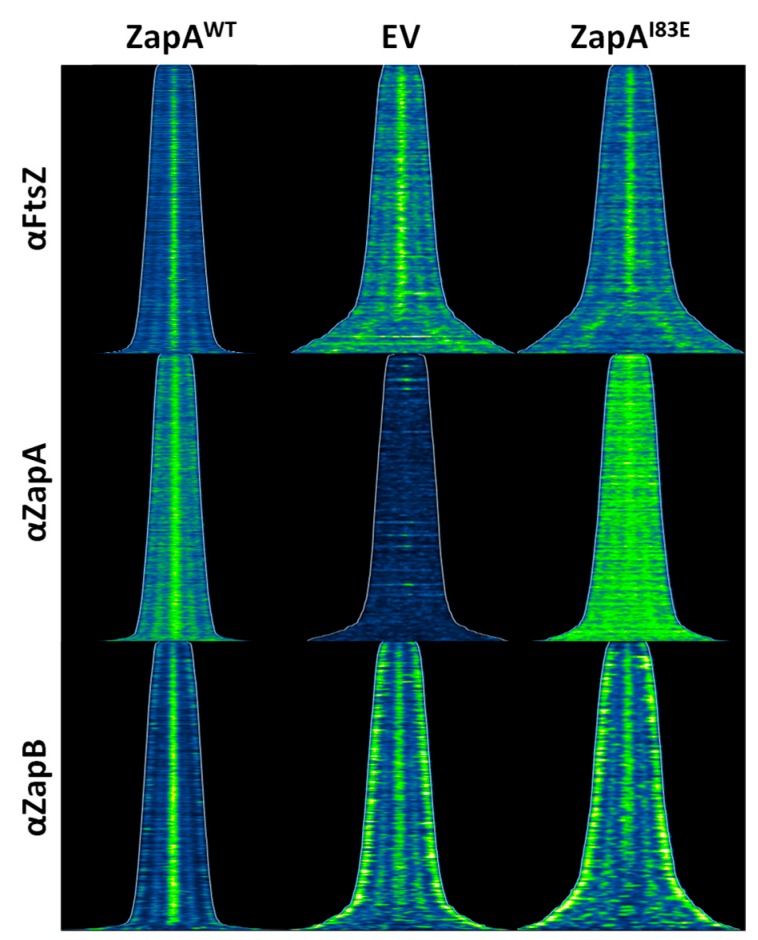
ZapA^I83E^ mainly localizes throughout the cytoplasm and does not complement ∆*zapA* cells based on the loss of specific Z-associated protein B (ZapB) localization. Maps of fluorescence profiles of TB28 ∆*zapA* cells expressing wild-type Z-associated protein A (ZapA), an empty vector control (EV) or ZapA^I83E^ immunolabeled with antibodies against filamenting temperature-sensitive Z (FtsZ), ZapA, or ZapB. Cells are sorted according to cell length using ObjectJ [17] and the fluorescence intensities of each labeling is shown at identical brightness and contrast values for direct comparison. Images of immunolabeled cells and average profiles along the cell cycle are presented in Figure S1.

ZapA^I83E^ was unable to complement a *zapA* deletion strain based on average cell length. In vitro work suggested that dimeric ZapA^I83E^ is still be able to bind FtsZ [6]. Therefore, it was hypothesized that ZapA^I83E^ would localize with FtsZ at midcell and that this may obstruct proper divisome functionality. The ∆*zapA* cells from the complementation experiment were immunolabeled with antibodies against FtsZ, ZapA, or ZapB and a fluorescent secondary antibody to probe their localization patterns. This revealed that ZapA^I83E^ localized mostly throughout the cell, apparently unable to fully bind FtsZ at midcell (Figure 2 and Figure S1). FtsZ localized mostly at midcell for all cultures but was also slightly more diffuse for the ∆*zapA* and the ZapA^I83E^ cells. Since ZapB midcell localization is dependent on ZapA [14], it was expected to also localize diffusely through the cells expressing ZapA^I83E^. Instead, ZapB also localized polarly in these cells mimicking the pattern of cells without ZapA. This sets ZapA^I83E^ apart from other non-complementing ZapA variants with mutations in the globular head domain that did localize to midcell [15] and recruited ZapB [7]. Interestingly, there is still a considerable midcell localization of ZapB in the cells without any wild-type ZapA that would not be expected if ZapA was the only recruiting factor [16]. The ZapB localization pattern is a good indication whether ZapA complements at midcell (Figure S2). 

### 2.3. ZapA^I83E^ Interacts with FtsZ In Vivo

The in vivo ZapA^I83E^ localization results did not seem to confirm the in vitro evidence that it still binds FtsZ [6]. However, its diffuse localization pattern does not exclude the possibility that it is still interacting with FtsZ. ZapA^I83E^ may interact with FtsZ that is therefore not able to polymerize as a part of the Z-ring. Indeed, the lack of FtsZ bundling was one of the conclusions of the in vitro work using ZapA^I83E^ [6]. This should not necessarily have major effects on division given the large amount of FtsZ in the cell of which only one third is involved in formation of the Z-ring. The critical concentration of FtsZ for polymerization in vitro is ~1 μM, whereas in vivo 5 μM is present. The amount of ZapA^I83E^ produced from plasmid is approximately that of the endogenous concentration of 1.6 μM as monomer [7,17]. Not only is there three times as much FtsZ as ZapA in the cell; FtsZ overexpression may compensate for division defects [18]. To assess the ZapA^I83E^–FtsZ interaction regardless of its localization pattern in vivo, a FRET experiment was attempted. 

**Figure 3:**
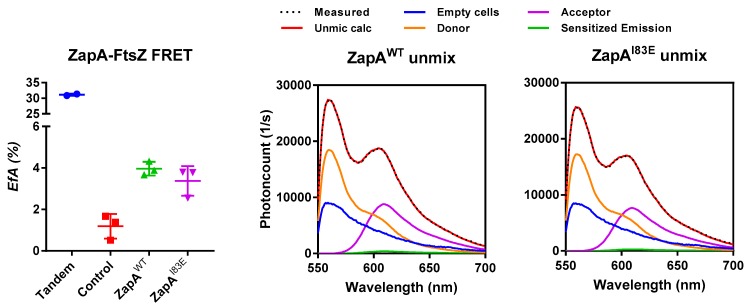
FtsZ and ZapA^I83E^ in vivo interaction FRET assay. On the left, the energy transfer efficiency of the FRET interactions indicated in the text. The positive and negative controls suggest a reliable experiment. On the right, typical emission spectra plotted against the photoncounts for the mCh–ZapA and mKO–FtsZ pairs. The contribution of the direct excitation of mCh (purple), mKO (orange), and the background autofluorescence (blue) to the measured spectrum (dotted line) were obtained by spectral unmixing [19,20]. The remaining signal (green) is the sensitized emission. mCh–ZapA^WT^ and mCh–ZapA^I83E^ showed similar fluorescence spectra with mKO–FtsZ resulting in similar E*f*A values.

The interaction between mKO–FtsZ and mCh–ZapA has been demonstrated in vivo by FRET with 4.5% energy transfer [19]. We aimed to compare the FtsZ–ZapA^WT^ interaction to the putative FtsZ–ZapA^I83E^ interaction. The FP-fusions to ZapA^WT^ and ZapA^I83E^ were confirmed to localize under the conditions of a FRET experiment and complement as described for the non-fused proteins. In addition, the presence of endogenous ZapA did not change the localization pattern of ZapA^I83E^ suggesting that the mutant will not form multimers in combination with ZapA^WT^ (Figure S3). For the FRET experiment the ∆*zapA* strain was used to increase the chances of plasmid-expressed mKO–FtsZ and mCh–ZapA interacting and the experiment was performed as described previously [19,20]. The direct fusion between mKO and mCh forming a tandem as positive control gave 31% energy transfer (E*f*A). The negative controls consisted of either mCh–ZapA, mCh–ZapA^I83E^, or mCh–PBP1b paired with the non-interacting inner membrane protein mKO–PBP1a and gave low E*f*A values of less than 1.7%. Together, these controls suggested a good detection range for the performed FRET experiments (Figure 3). Wild-type mCh–ZapA interacted with mKO–FtsZ showing the expected energy transfer efficiency of 4%. Interestingly, mCh–ZapA^I83E^ gave very similar E*f*A values and fluorescence spectra, underscoring equal expression. The FRET experiments confirmed the interaction of ZapA^I83E^ with FtsZ previously shown in vitro [6]. The FRET experiment was repeated in the WT strain resulting in similar E*f*A values of 4.5% and 5.5% for ZapA and ZapA^I83E^, respectively.

### 2.4. ZapB Delocalizes Unipolarly in Cells with ZapA^I83E^ or Without ZapA

**Figure 4:**
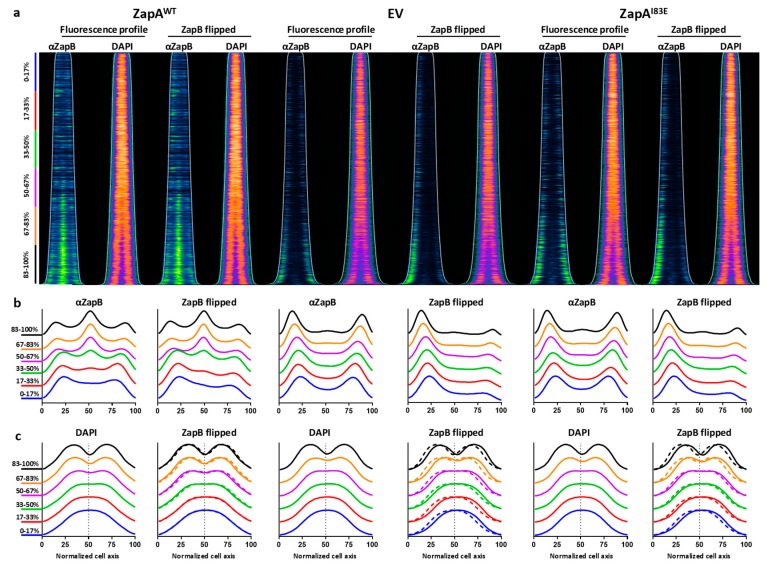
ZapB localized unipolarly in Δ*zapA* or with ZapA^I83E^ expressed from plasmid and was anti-correlating with the chromosomal position in cells grown to steady state in minimal medium. (**a**) Localization profiles of immunolabeled ZapB and DAPI-stained nucleoids in Δ*zapA-*expressing ZapA^WT^, EV, or ZapA^I83E^. The ZapB profiles showed strong signals at midcell in cells with functional ZapA and polar localization in cells without it or with the mutant ZapA^I83E^. See Figure 2 for comparison to ZapB localization in cells grown under rich medium conditions. When the cells were sorted according to length and with the strongest polar ZapB signal on the left side of the cell (flipped), predominantly unipolar signals were observed. The DAPI signal in these cells seemed to localize away from ZapB. (**b**,**c**) The flipped and non-flipped ZapB and DAPI profiles were grouped as 17% age bins and plotted. The DAPI signal in cells without ZapA or with ZapA^I83E^ seemed to have shifted towards the middle of the cells, away from the polar ZapB and showed less invagination (Figure S4). The dashed DAPI profiles represent the mirrored localization and emphasize the changes in chromosome localization patterns. The respective number of analyzed ZapA^WT^, EV, and ZapA^I83E^ cells were 1089, 3213 and 2105.

ZapB localizes at midcell in wild-type cells but mainly polarly in the absence of ZapA [7,13,21]. In the presence of diffusely-localized ZapA^I83E^, ZapB also strongly localizes polarly instead of following the diffusely-distributed pattern. To investigate, TB28 ∆*zapA* containing either *zapA^WT^*, z*apA^I83E^* on plasmids, or an empty vector (EV) negative control were grown to steady state in minimal medium to correlate cell length to cell age over the cell cycle without large differences in cell size [17] (Figure S4). Expression was induced with 50 µM IPTG for at least two mass doublings and the cells were fixed and harvested for immunolabeling with antiZapB before imaging and analysis. The resulting fluorescence profiles showed the expected ZapB midcell localization in the cells expressing ZapA^WT^ and mainly polar localization for cells expressing ZapA^I83E^ or EV (Figure 4). The ObjectJ “poleflipper” macro was used to orient the cell profiles based on the localization of the strongest ZapB signal [17]. This revealed striking unipolar ZapB signals for the cells expressing ZapA^I83E^ or EV and an average midcell localization for the cells expressing ZapA^WT^ (Figure 4b). The polar localization of ZapB strongly suggests that ZapA^I83E^ is unable to interact with ZapB. 

### 2.5. Unipolar ZapB Signal Anticorrelates with Signal for the Chromosome

ZapB interacts with MatP, which connects the chromosomal *ter* region to the divisome [10]. Therefore, this link may be severed if ZapB is delocalized and the position or orientation of the chromosome may be affected. To test this, the nucleoids of the ∆*zapA* cells expressing ZapA^WT^ or ZapA^I83E^ from plasmid or harboring an EV were visualized with DAPI (Figure 4). When the ZapB poles are flipped, it became apparent that the nucleoid signal anti-correlated with the ZapB signal. In the ZapB pole-flipped DAPI profiles for EV and ZapA^I83E^ the nucleoids were localized away from ZapB while for cells with ZapA^WT^ the DAPI signal was more symmetrically distributed (Figure 4bc). Overlaying the DAPI fluorescence profiles shows that the nucleoids were more localized towards midcell and started to invaginate less prominently and later in the cell cycle for cells without ZapA or with the in vitro non-tetramerizing mutant ZapA^I83E^ (Figure S4b). Cells expressing ZapA^WT^ showed deeper nucleoid invagination at the site where cell division requires proper nucleoid segregation (Figure S4bc). In fact, it appears that constriction itself was also shifted off midcell suggesting asymmetric division in cells with unipolar ZapB (Figure S4cd). The interaction partner of ZapB, MatP, indeed concentrates the *ter* region of the chromosome and a severed *ter* linkage may influence localization and compaction during cell division [11]. This may explain why the DAPI signals showed less chromosome invagination in cells with unipolar ZapB. These observations may provide further insights in *ter* linkage through ZapB function and chromosome segregation. The question remains whether the affected chromosome localization is a direct effect of polar ZapB. It is possible that the nucleoids were merely occupying the available space or that other factors direct the chromosome away from the pole with ZapB.

### 2.6. Min Oscillation Is Not Affected by Polar ZapB, Polar ZapB Is Likely Folded and Functional

**Figure 5:**
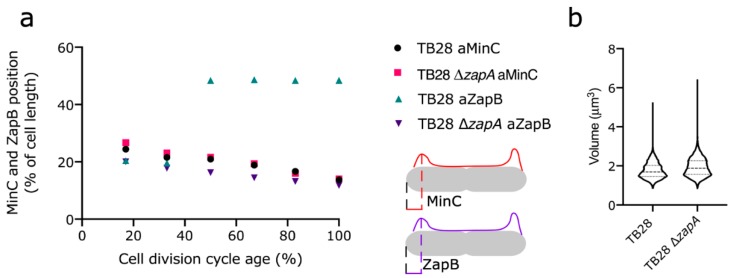
MinC localization was identical in TB28 and ∆*zapA* despite the polar presence of ZapB in the latter strain. (**a**) The distance from the ZapB containing cell pole to the position of MinC in that cell pole was determined in TB28 ∆*zapA* and compared with the position of MinC in wild-type cells and plotted against the normalized cell division cycle age (see also cartoon). The position of ZapB in the pole with the strongest ZapB signal in TB28 ∆*zapA* was also plotted and compared to its position in the wild-type strain. In the wild-type strain, all ZapB molecules ended up at midcell, whereas in the ∆*zapA* strain only a minor amount was present at midcell (see Figure S5 for complete profiles). (**b**) Volume distribution of TB28 and TB28 ∆*zapA* cells. The number of cells analyzed for TB28 a(anti)MinC, TB28 ∆*zapA* aMinC, TB28 aZapB, and TB28 ∆*zapA* aZapB were 3201, 4154, 3473, and 1726, respectively.

To prove that ZapB is not an inclusion body that is simply obstructing a pole, but is still dynamic, the ∆*zapA* strain and its parental TB28 wild-type strain were immunolabeled with antiMinC. Min proteins oscillate from pole to pole where they inhibit Z-ring formation and by a concentration-dependent manner allow the proto-ring to form at midcell. Previously, it was shown that the localization pattern of immunolabeled MinC reflects its oscillatory behavior [17]. The MinC pattern was identical in both strains, indicating that MinC completely ignored the presence of ZapB with respect to its position in the cell (Figure 5). These data suggest that the polar ZapB is properly folded and delocalized solely because its midcell recruiting partner ZapA is missing or affected by the I83E mutation. Indeed, counter-oscillation of FtsZ, ZapA, and ZapB has been proposed to build up the new cell division site influenced by Min [21]. The in vitro non-tetramerizing mutant ZapA^I83E^ and ZapA mutants that are affected in their ability to interact with FtsZ localize more diffusely in the cytoplasm [7]. Therefore, they seem not to oscillate despite the normal oscillation of the Min system (Figure S5).

### 2.7. ZapA and MatP Effects on ZapB Localization

**Figure 6:**
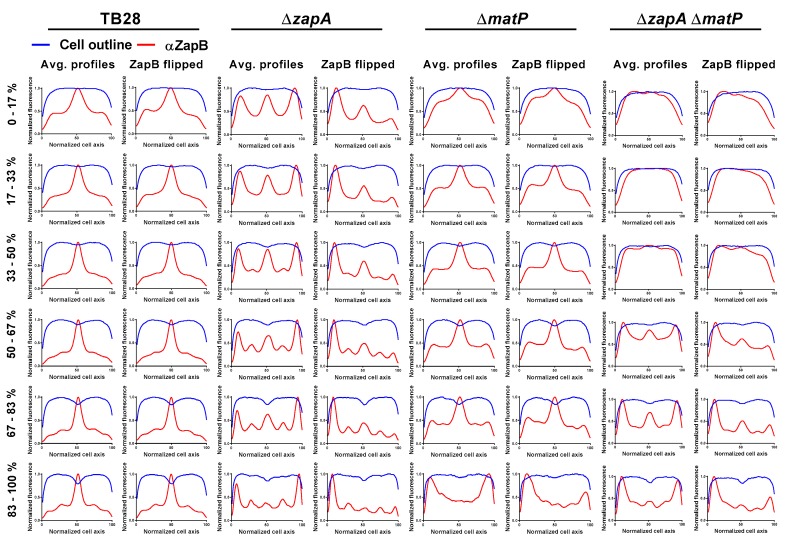
ZapA is the main factor affecting ZapB. localization at midcell. The cells were grown in rich medium at 37 °C, fixed, and immunolabeled with antiZapB. Average fluorescence profiles (red) and diameter profiles (blue) in 17% age classes of TB28, ∆*zapA*, ∆*matP* or ∆*zapA* ∆*matP* are shown from left to right, respectively. For each sample the random orientation and the ZapB intensity-based orientation are shown. Absence of MatP had minor effects on ZapB midcell localization but ZapB signals localized more throughout the cell. The full fluorescence profile maps are shown in Figure S6. The respective number of analyzed cells for TB28 ∆*zapA*, ∆*matP* or ∆*zapA* ∆*matP* were: 1504, 906, 759, and 831.

Both ZapB and MatP are required to bring *matS* sites (and thus *ter*) to the division site [22]. If the physical interaction of ZapB and MatP is required for the correct localization of the chromosomes, changes in ZapB dynamics are expected in a ∆*matP* strain. Strain TB28 and ∆*zapA*, ∆*matP,* or ∆*zapA* ∆*matP* in an isogenic background were grown under rich medium conditions before they were fixed, harvested, and labeled with antiZapB. The fluorescence profiles were analyzed using ObjectJ (Figure 6). As expected, ZapB localized at midcell in the wild-type cells and mainly (uni)polar in the ∆*zapA* cells. In the absence of MatP, ZapB was still able to be recruited to midcell but also partly localized to the poles and in the cells’ periphery. The ZapB localization signals were substantially stronger in cells without MatP suggesting either more ZapB production by the cells or that more ZapB epitope became available to antiZapB. The double-deletion strain showed the combined effects of the single *zapA* or *matP* deletions. In these cells, ZapB localized mainly polar but also more throughout the cell. These results support the idea that both ZapA and MatP influence the localization of ZapB. In such a model ZapA has a role in guiding ZapB to midcell and MatP helps in keeping ZapB from freely distributing throughout the cell. 

## 3. Discussion and Conclusions

ZapA is an FtsZ-associated protein involved in stabilization of the proto-ring and synchronizing bacterial cell division with chromosome segregation through *ter* linkage. ZapA can exist as tetramers or dimers and in vitro evidence suggests its functionality relies on its tetrameric form, even though the dimer can still bind to FtsZ [5,6]. Our work investigated the in vivo effects of a ZapA mutant that is unable to form tetramers in vitro (ZapA^I83E^) in relation to cell division through FtsZ and in relation to chromosome segregation through ZapB. The ZapA^I83E^ mutant was designed to disrupt the multimerization interface and yield parallel ZapA dimers with the globular head group intact to allow FtsZ dimer binding [6,7]. Indeed, previous in vitro and now in vivo work show that ZapA^I83E^ is able to interact with FtsZ. However, the localization pattern of ZapA^I83E^ also shows that most of it is not attached to the FtsZ proto-ring. This suggests that ZapA^I83E^ is only able to interact with free cytosolic FtsZ that may therefore be incapable of polymerizing into the proto-ring. Alternatively, ZapA^I83E^ may be unable to bind dynamic FtsZ filaments. Although the ability of ZapA^I83E^ to form tetramers in vivo was not directly investigated, it was shown to be non-functional in a complementation assay. Therefore, it is tempting to speculate that ZapA tetramerization is required for its midcell localization and subsequent interactions and that ZapA^I83E^ could be used as a tool to investigate the effects of dimeric ZapA.

Another remaining question is whether ZapA stoichiometry or its interaction with FtsZ at midcell is required for ZapB midcell localization. ZapA mutants in its globular headgroup (R13D, E51K, and I56K) were found to be not complementing cell length (>10% filamentous cells) but still localized at midcell, as did ZapB [7]. This suggested they were interacting with FtsZ and independently still recruited ZapB, apparently affecting the proto-ring in a different manner. The recently-published non-complementing ZapA^R46A^ mutation that has been shown to tetramerize but not bind FtsZ may help to answer this question [5,9].

ZapB becomes predominantly polar in cells without ZapA or with ZapA^I83E^ and nucleoid localization is affected. When ZapB’s other interaction partner MatP is absent, more ZapB is detectable and is still able to localize to midcell but also localizes diffusely and in the cell poles. When both interaction partners are missing, ZapB’s localization pattern becomes even more diffuse and more bipolar. This suggests a role for MatP in the unipolar localization of ZapB in ∆*zapA* cells and continued dynamics when the *ter* linkage is broken. Interestingly, the chromosomal localization patterns anticorrelate to that of ZapB and, although speculatively, polar ZapB may have a function in WT cells at the beginning of the cell cycle. In this case, ZapB is part of the division ring (i.e., not aggregating) and may repulse the DNA after division completes and a new cycle commences. The negative charge of ZapB may be of influence on chromosome localization considering its high abundance in the cell [23] with its 17 negative and 8 positive residues this could be significant. However, presently no evidence exists for direct effects of ZapB on the chromosome. In conclusion, the in vitro dimeric ZapA mutant ZapA^I83E^ cannot bind FtsZ protofilaments in the Z-ring at midcell and fails to interact with ZapB, which results in asymmetric nucleoid organization and division.

## 4. Materials and Methods 

### 4.1. Bacterial Strains and Growth Conditions

*Escherichia coli* K12 strains used in this work are presented in Table 1. The cells were cultured in rich medium (TY: 10 g tryptone (Bacto Laboratories, Australia), 5 g yeast extract (Duchefa, Amsterdam, The Netherlands) and 5 g NaCl (Merck, Kenilworth, NJ, USA) per liter), supplemented with 0.5% glucose (Merck) or in glucose minimal medium (Gb1: 6.33 g K_2_HPO_4_ (Merck), 2.95 g KH_2_PO_4_ (Riedel de Haen, Seelze, Germany), 1.05 g (NH_4_)_2_SO_4_ (Sigma, St. Louis, MO, USA), 0.10 g MgSO_4_·7H_2_O (Roth, Karlsruhe, Germany), 0.28 mg FeSO_4_·7H_2_O (Sigma), 7.1 mg Ca(NO_3_)_2_·4H_2_O (Sigma), 4 mg thiamine (Sigma), and 4 g glucose per liter, pH 7.0) at 28 °C while shaking at 205 rpm. For growth of TB28-based strains in Gb1, 50 mg lysine, 50 mg arginine, 50 mg glutamine, 20 mg uracil, and 2 mg thymidine (all from Sigma), were added per liter. Expression of protein was induced with isopropyl β-D-1-thiogalactopyranoside (IPTG, Promega, Madison, WI, USA) as indicated. Plasmids were maintained in the strains by addition of 100 µg mL^−1^ ampicillin (Sigma) or 25 µg mL^−1^ chloramphenicol (Sigma). Growth was measured by absorbance at 600 or 450 nm with a Biochrom Libra S70 spectrophotometer (Harvard Biosciences, Holliston, MA, USA) for TY or Gb1 cultures, respectively. TB28 ∆*matp* and TB28 ∆*zapA* ∆*matp* were created by P1 transduction of *matp::kan* from the KEIO collection [24] into TB28 and TB28 ∆*zapA,* respectively. The presence of the deletions was confirmed by PCR.

### 4.2. Site-directed Mutagenesis and Plasmid Construction

The plasmids used in this study are shown in Table 2. Plasmid pGP021 [3] expresses ZapA from a weakened *trc99A* promotor, which is IPTG inducible. The I83E mutation was introduced in the ZapA-expressing plasmid pGP016 (6HisZapA) using the Quick change mutagenesis method (Stratagene, la Jolla, CA, USA). The used primers were 5′-GTATGGAACAGCGTGAACGGATGCTGCAGC-3′ and 5′-GCTGCAGCATCCGTTCACGCTGTTCCATAC-3′ and the mutagenesis resulted in plasmid pET302His6ZapA^I83E^. From this plasmid *zapA^I83E^* was cut using NcoI and HindIII and inserted in pTHV037 to yield plasmid pRP071. Plasmid mCh–ZapA^I83E^, pNM137, was created by exchanging *zapA* in pSAV077 with *zapA^I83E^* from pRP071 by restriction digestion cloning using DraIII and HindIII. 

### 4.3. Immunolabeling

Immunolabeling of cells was performed as described in [29] with antibodies against ZapA, FtsZ, ZapB, or MinC [17]. The ZapA antiserum was routinely purified by adsorption against TB28 Δ*zapA* cells to filter out potential cross-reactive IgG. The supernatant was subsequently used to label the ZapA mutants.

### 4.4. Microscopy and Image Analysis

For imaging, the cells were immobilized on 1% agarose [30] and photographed with a CoolSnap fx (Photometrics) charge-coupled device (CCD) camera mounted on an Olympus BX-60 fluorescence microscope through an UPLANFl 100×/1.3 oil objective (Olympus, Tokyo, Japan). Images were taken using modified acquisition software that used the program ImageJ by Wayne Rasband and analyzed using Object-J’s Coli-Inspector [17]. Statistical testing of normalized DAPI localization patterns was done using Kolmogorov–Smirnov testing available on http://www.physics.csbsju.edu/stats/ [31].

### 4.5. FRET Assay

The FRET experiments were performed as originally described in [19,20] with the plasmids shown above. 

## Figures and Tables

**Table 1:** Strains used.

Name	Full Name	Reference
**DH5α**	*F^-^*, *supE44*, *hsdR17*, *recA1*, *endA1*, *gyrA96*, *thi1*, *relA1*	[25]
**TB28**	*rph1 ilvG rfb-50 ΔlacIYZA::FRT*	[26]
**TB28 ∆*zapA***	TB28 ∆*zapA*	[14]
**sNM02**	TB28 ∆*matP*	This work
**sNM03**	TB28 ∆*zapA* ∆*matP*	This work

**Table 2:** Plasmids used in this study.

Name	Full Name/Characteristics	Reference
**pTHV037**	p*trc*99Adown, AmpR, ColE1 ori	[27]
**pSAV057**	p*trc*99Adown, CamR, P15 ori	[19]
**pGP021**	pTHV–ZapA	[3]
**pGP016**	pET302His6ZapA, p*trc*99A, AmpR, ColE1 ori	[3]
**pET302His6ZapA^I83E^**	pET302His6ZapA^I83E^	This work
**pRP071**	pTHV–ZapA^I83E^	This work
**pSAV050**	pTHV–mCh–mKO	[19]
**pSAV072**	pSAV–mKO–FtsZ	[19]
**pSAV077**	pTHV–mCh–ZapA	[19]
**pNM137**	pTHV–mCh–ZapA^I83E^	This work
**pBB008**	pTHV–mCh–PBP1b	[28]
**pBB004**	pSAV–mKO–PBP1a	[28]

## Supplementary Materials

Reference [32] is cited in the supplementary materials. Supplementary materials can be found at https://www.mdpi.com/1422-0067/21/9/3130/s1.
