# Supplementary material for: The In Vitro Non-Tetramerizing ZapAI83E Mutant Is Unable to Recruit ZapB to the Division Plane In Vivo in Escherichia coli
Source: Int J Mol Sci. 2020 Apr 29;21(9):3130. doi: 10.3390/ijms21093130 (PMC7246612; doi:10.3390/ijms21093130)
Supplement: Supplementary file 1 [file ijms-21-03130-s001.pdf]

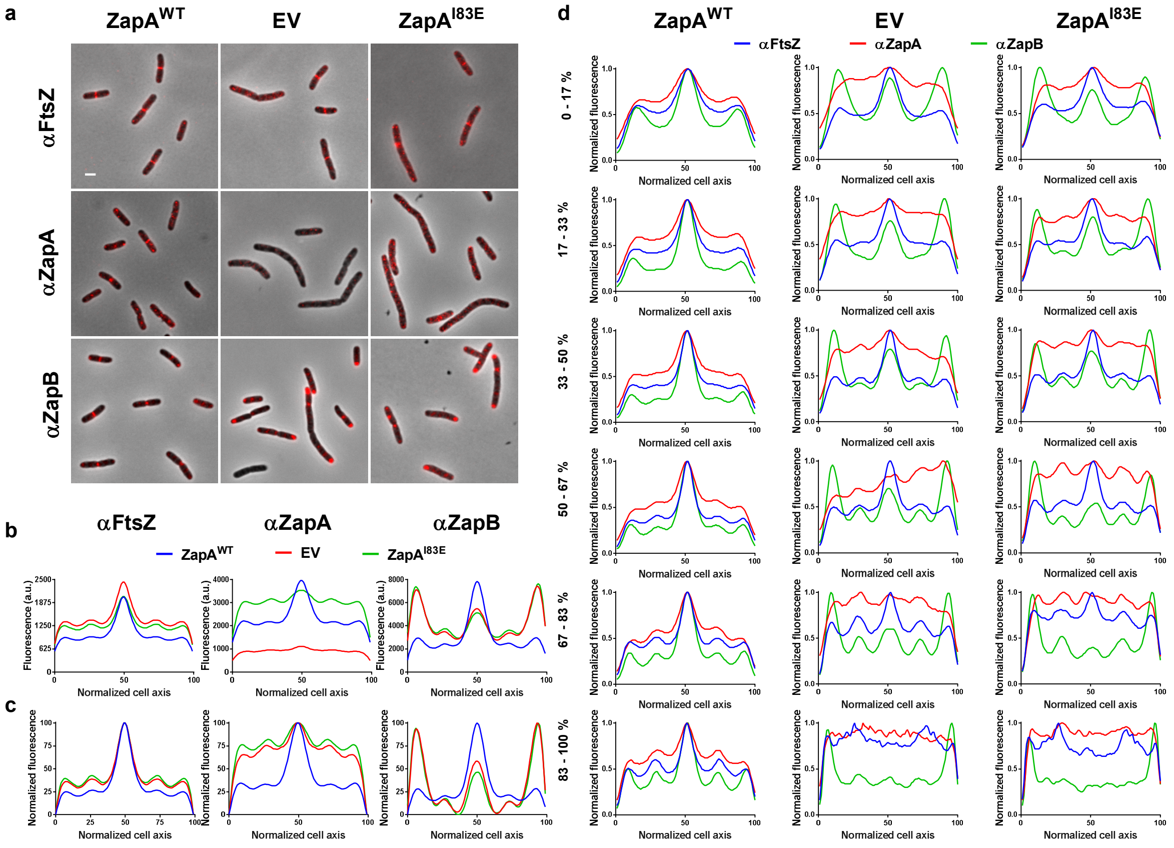

Figure S1. ZapA^{I83E} did not localize mainly at midcell and did not complement based on ZapB localization. (**a**) TB28 Δ*zapA* cells expressing ZapA^{WT}, an EV control or ZapA^{I83E} were immunolabeled with antiFtsZ, antiZapA, and antiZapB and a fluorescent secondary antibody (red). The fluorescence signals from the cells were imaged and analyzed using ObjectJ [17]. The fluorescence intensities are shown at identical brightness and contrast values and the scale bar represents 2 µm. (**b**,**c**) The average fluorescence profiles (**b**) and the peak normalized average fluorescence profiles (**c**) along the normalized cell axis revealed FtsZ midcell localization for all groups. ZapA^{I83E} was expressed and detectable and followed the localization distribution pattern of the EV negative control. Consequently, strong ZapB midcell localization was not observed for ZapA^{I83E} and EV. (**d**) Normalized fluorescence profiles averaged based on 17%-cell-length classes. Maps of the fluorescence profiles are shown in Figure 2.

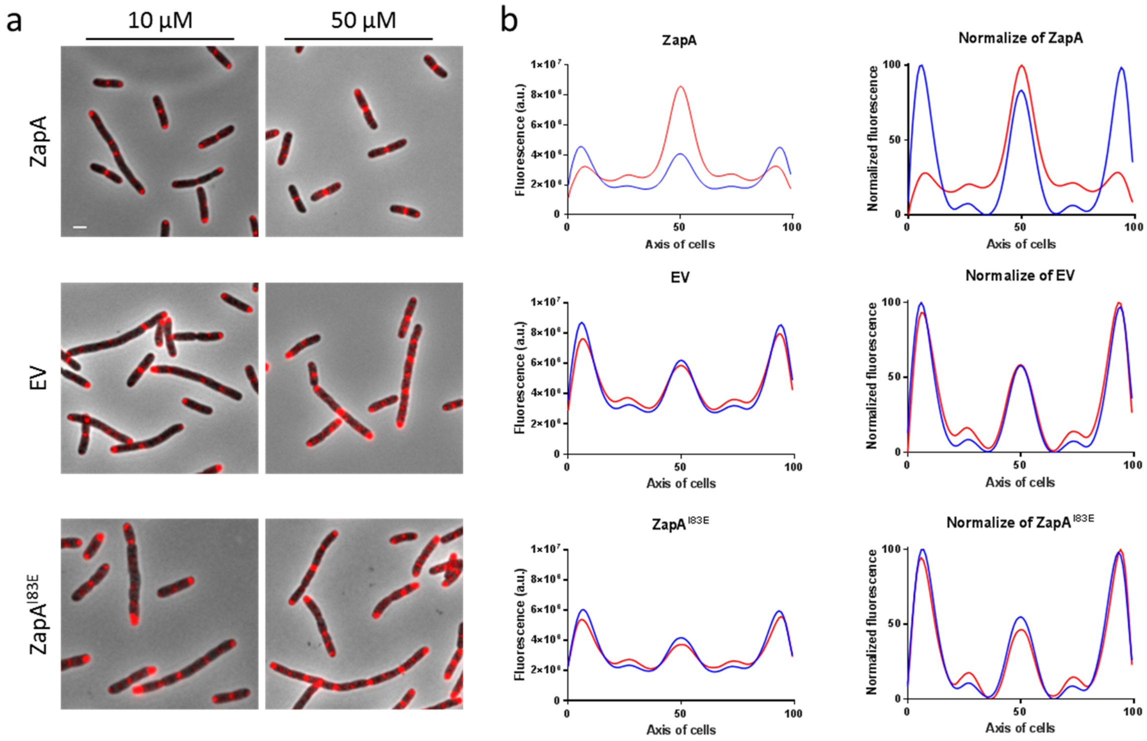

Figure S2. ZapB midcell localization was an indicator for ZapA complementation. (**a**) At low induction concentrations ZapA function was not (fully) complemented, the cells became longer and ZapB localized strongly in the cell poles even for the Δ*zapA* strain transformed (but weakly induced) with ZapA^{WT}. When induced with 50 µM IPTG, ZapA^{WT} from plasmid was able to complement the deletion phenotype resulting in wild type cell lengths and strong ZapB localization at midcell. The negative control of the Δ*zapA* strain transformed with EV did not complement the deletion phenotype at either induction concentration, the cells remained longer and ZapB localized strongly in the cell poles. The in vitro non-tetramerizing ZapA mutant I83E was also unable to complement the Δ*zapA* phenotype and ZapB followed the same pattern as in the EV cells suggesting it is non-functional in terms of cell length complementation and ZapB midcell localization. The scale bar represents 2 µm. (**b**) The average ZapB profiles of the 10 and 50 µM IPTG-induced cells are shown by blue lines and red lines, respectively.

ZapA^{WT} and ZapA^{I83E} fusions do not further obstruct functionality

The mCh–ZapA^{WT} and mCh–ZapA^{I83E} constructs were tested for their use in a FRET experiment. First, their ability to complement the Δ*zapA* phenotype under minimal medium conditions was assessed. The mCh–ZapA^{WT} fusion complemented the cell length in Δ*zapA* strain with average cell lengths of 2.9 µm, which were very similar to the WT cells expressing the same construct. mCh–ZapA^{I83E} did not complement the *zapA* deletion strain and resembled the average cell length of the EV control cells more, with 3.2 µm.

Localization patterns were observed in wild-type as well as in Δ*zapA* cells to ascertain whether chromosomal expression of ZapA would influence the localization of ZapA^{I83E}. Additionally, this could indicate whether mCh-ZapA^{I83E} would be able to tetramerize with WT ZapA and potentially localize at midcell. mCh–ZapA^{WT} accumulated at midcell during cell division as was indicated by its increasing FCPlus (surplus of fluorescence in cell center compared to the rest of the cell [17]) as a function of cell age, suggesting that it was functional (Figure S3). The mCh–ZapA^{I83E} mutant was more distributed throughout the cell. The fluorescence localization pattern of mCh–ZapA^{I83E} was identical in the WT and Δ*zapA* strain. This makes is it less likely that wild-type ZapA and ZapA^{I83E} were able to form tetramers together.

mCh–ZapA^{WT} was able to recruit ZapB to midcell in WT as well as in Δ*zapA* cells underscoring its functionality albeit the ZapB signal was less pronounced. The latter observation may suggest that the FP fusion could be less favorable for ZapB binding. mCh–ZapA^{I83E} did not allow ZapB localization at midcell, completely mimicking the *zapA* deletion strain. ZapB localization in the WT strain was unhindered by the expression of ZapA^{I83E}. This again suggests that ZapA^{WT} and ZapA^{I83E} do not form multimers together. It was concluded that the constructs could be used for a FRET experiment assaying their interaction with FtsZ.

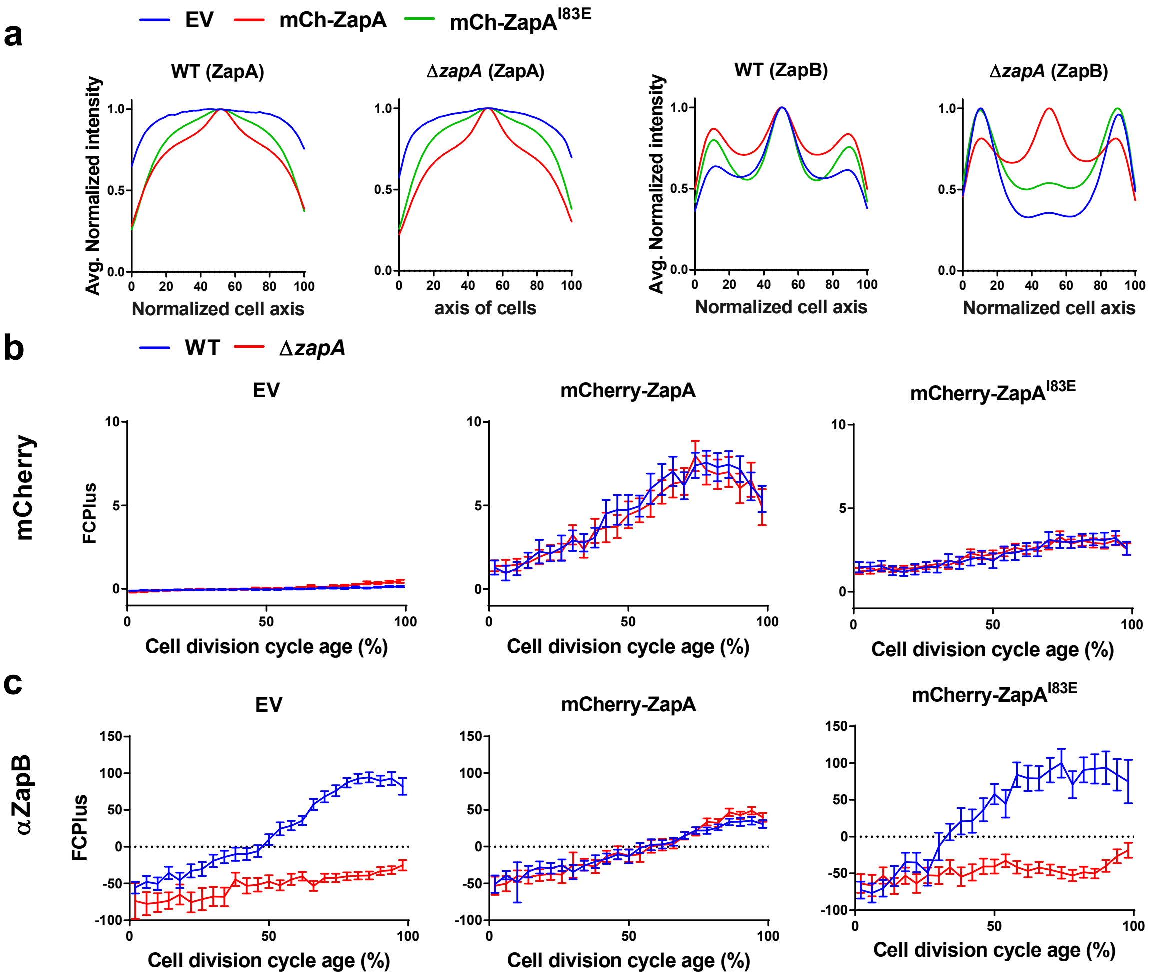

Figure S3. mCh–ZapA independently localized to midcell and recruited ZapB but mCh-ZapA^{I83E} did not show strong affinity for midcell and had lost the ability to recruit ZapB. (**a**) Localization patterns of mCh–ZapA and mCh–ZapA^{I83E} in WT and Δ*zapA* cells (ZapA) and their ZapB immunolabeling (ZapB). (**b**) mCh FCPlus values of steady-state-growing TB28 and TB28 Δ*zapA* cells expressing mCh–ZapA, mCh–ZapA^{I83E} or carrying the EV control. FCPlus is the extra fluorescence in a 400 nm rectangle at mid cell in comparison to the rest of the cell [17]. In both the WT and Δ*zapA* strain mCh-ZapA midcell concentrations increased as the cell division cycle progressed reaching the maximum concentration at around 70%–80 %. mCh–ZapA^{I83E} showed a less pronounced midcell. (**c**) The same cells as for the mCh measurement were immunolabeled with antiZapB showing the WT–ZapB response on mCh–ZapA or the mutant. In the WT cells ZapB localized maximally at midcell around 80% of the cell cycle while in the Δ*zapA* strain it kept a polar fraction and was not able to bind ZapA at midcell anymore with the resulting negative FCPlus values. Expression of mCh–ZapA enabled ZapB to localize at midcell but mCh–ZapA^{I83E} did not. FCPlus values are plotted along the cell division age and binned into 4% age groups. The bar represents the 95% confidence interval. Between 1000 and 5000 cells were analyzed for each group.

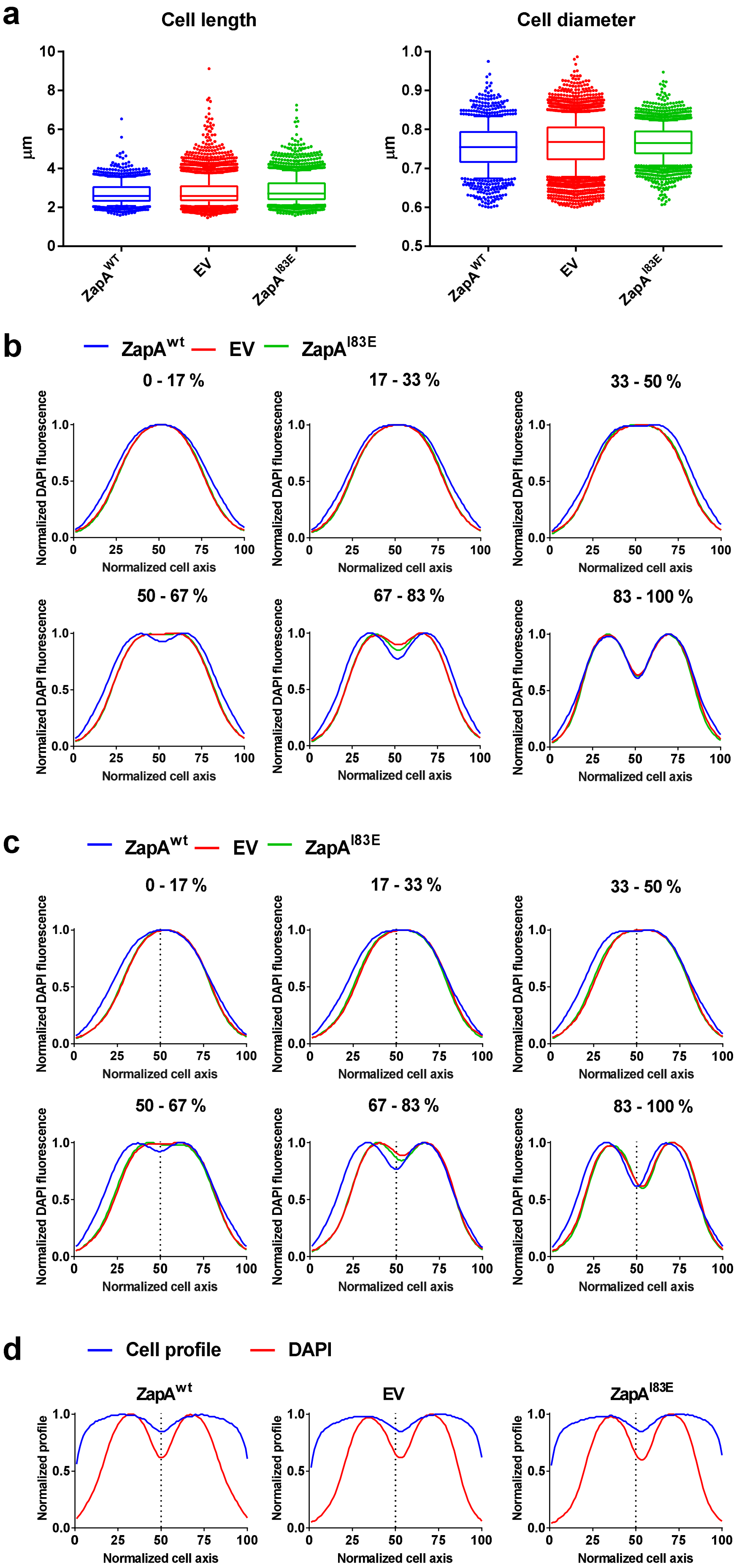
a
Cell length
µm
ZapA WT
EV
ZapA I83E
Cell diameter
b
ZapAwt
EV
ZapAI83E
0 - 17 %
17 - 33 %
33 - 50 %
50 - 67 %
67 - 83 %
83 - 100 %
Normalized DAPI fluorescence
Normalized cell axis
c
d
Cell profile
DAPI
Normalized profile

Figure S4. Cell lengths and diameters of the ZapB- and DAPI-labeled cells are presented in Figure 4. TB28 Δ*zapA* cells were grown at semi-steady state in Gb1 at 28 °C and expression of ZapA^{WT}, ZapA^{I83E}, or EV (empty vector control) was induced for at least two mass doublings with 50 μM IPTG. The cells were fixed and immunolabeled with antibodies against ZapB and a fluorescent secondary antibody. The chromosomes were visualized by DAPI staining. (**a**) The average cell lengths (in μm) were for ZapA^{WT}, 2.72; EV 2.79 and ZapA^{I83E}, 2.88 and the respective number of measured cells were 1089, 3213, and 2105. The whiskers represent the 10th and 90th percentiles. (**b**) Normalized average fluorescence profiles in 17%-cell-length classes of the nucleoids stained with DAPI for cells expressing ZapA (blue), the mutant ZapA I83E (green), or containing an empty vector (EV, red). (**c**) The ZapB-flipped profiles shown in b, ZapA (blue), the mutant ZapA I83E (green), or EV (red) suggesting asymmetric nucleotide distributions. The normalized intensity of the DAPI distributions were compared between wild type and EV and confirmed to be significantly different by a Kolmogorov–Smirnov test (D=0.1098, P=0.004), whereas the localization pattern between ZapA^{I83E} and EV was not significantly different (D=0.0499, P=0.55). (**d**) The invaginations of the cell outline profile (blue) and the nucleoid profiles (red) occurred at the same place in along the cell axis showing that constriction follows the asymmetric pattern of the nucleoids (83%–100% age groups shown).

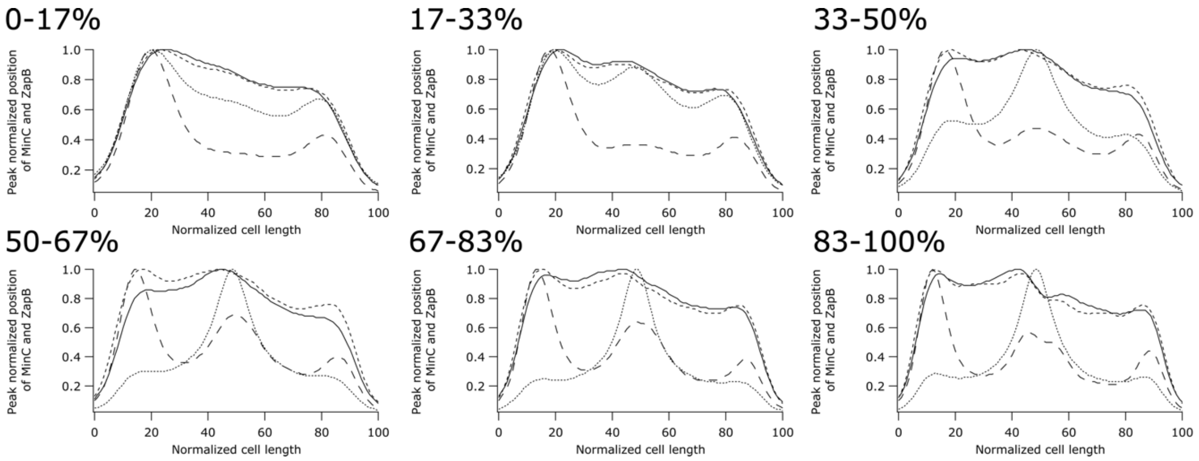

Figure S5. MinC was unaffected by the presence of ZapB with respect to is oscillation position in the Δ*zapA* cells. Peak-normalized binned average profiles of ZapB and MinC in the indicated age classes are plotted against the normalized cell length. The cells were grown in AB medium supplemented with glucose as carbon source [32]. Solid lines, TB28 antiMinC; dashed lines, Δ*zapA* antiMinC; dotted lines, TB28 antiZapB; and larger dash lines, Δ*zapA* antiZapB.

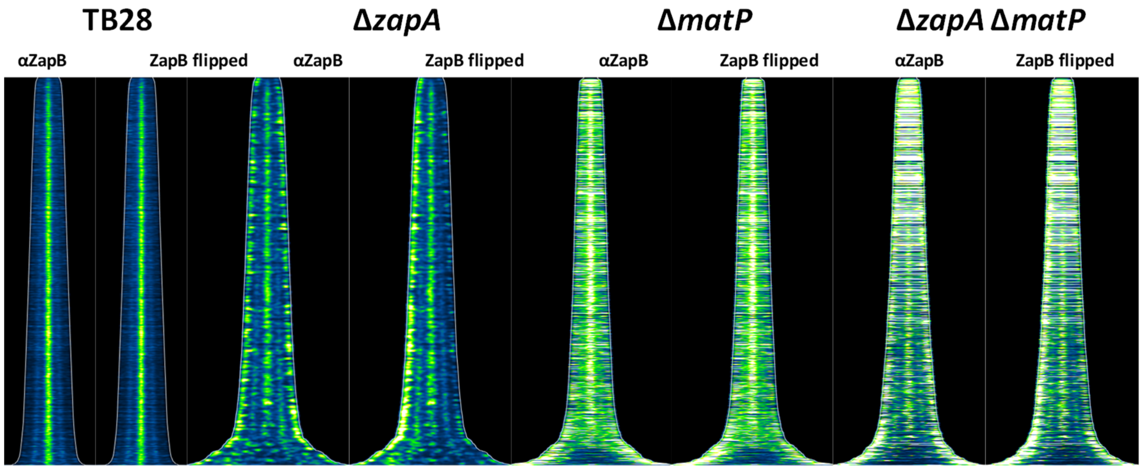

Figure S6. Map of fluorescence profiles of TB28, Δ*zapA*, Δ*matP*, or Δ*zapA* Δ*matP* cells immunolabeled with antibodies against ZapB and its flipped counterparts. Age-binned profiles are shown in Figure 6. The respective number of analyzed cells for TB28 Δ*zapA*, Δ*matP*, or Δ*zapA* Δ*matP* were: 1504, 906, 759, and 831.
